# Molecular epidemiology of SARS‐CoV‐2 in Mongolia, first experience with nanopore sequencing in lower‐ and middle‐income countries setting

**DOI:** 10.1002/iid3.1095

**Published:** 2023-12-13

**Authors:** Munkhtuya Erendereg, Suvd Tumurbaatar, Otgonjargal Byambaa, Gerelmaa Enebish, Natsagdorj Burged, Tungalag Khurelsukh, Nomin‐Erdene Baatar, Badmaarag Munkhjin, Jargaltulga Ulziijargal, Anuujin Gantumur, Oyunbaatar Altanbayar, Ochbadrakh Batjargal, Delgermurun Altangerel, Khosbayar Tulgaa, Sarangua Ganbold, Odgerel Tundev, Sarantsetseg Jigjidsuren, Tsogbadrakh Nyamdorj, Naranzul Tsedenbal, Bumdelger Batmunkh, Baigalmaa Jantsansengee, Battur Lkhagvaa, Bilegtsaikhan Tsolmon, Oyunsuren Enebish, Erdembileg Tsevegmid, Enkhbold Sereejav, Khurelbaatar Nyamdavaa, Ryenchindorj Erkhembayar, Battogtokh Chimeddorj

**Affiliations:** ^1^ Department of Microbiology and Infection Prevention Control, School of Biomedicine Mongolian National University of Medical Sciences Ulaanbaatar Mongolia; ^2^ Intermed Hospital Ulaanbaatar Mongolia; ^3^ Institute of Biomedical Sciences Mongolian National University of Medical Sciences Ulaanbaatar Mongolia; ^4^ National Center for Zoonotic Disease Ulaanbaatar Mongolia; ^5^ National Center for Public Health Ulaanbaatar Mongolia; ^6^ Division for Science and Technology Mongolian National University of Medical Sciences Ulaanbaatar Mongolia; ^7^ School of Medicine Mongolian National University of Medical Sciences Ulaanbaatar Mongolia; ^8^ National Center for Communicable Diseases Ulaanbaatar Mongolia; ^9^ First Central Hospital of Mongolia Ulaanbaatar Mongolia; ^10^ Ministry of Health Ulaanbaatar Mongolia; ^11^ Mongolian National University of Medical Sciences Ulaanbaatar Mongolia; ^12^ International Cyber Education Center, Graduate School Mongolian National University of Medical Sciences Ulaanbaatar Mongolia; ^13^ Department of Global Health and Population Harvard T.H. Chan School of Public Health Boston Massachusetts USA

**Keywords:** COVID‐19, mongolia, SARS‐CoV‐2, molecular epidemiology, whole genome sequencing

## Abstract

**Background:**

Coronavirus disease (COVID‐19) has had a significant impact globally, and extensive genomic research has been conducted on severe acute respiratory syndrome coronavirus 2 (SARS‐CoV‐2) lineage patterns and its variants. Mongolia's effective response resulted in low prevalence until vaccinations became available. However, due to the lack of systematically collected data and absence of whole genome sequencing capabilities, we conducted a two‐stepped, nationally representative molecular epidemiologic study of SARS‐CoV‐2 in Mongolia for 2020 and 2021.

**Methods:**

We used retrospective analysis of stored biological samples from November 2020 to October 2021 and a variant‐specific real‐time reverse transcription polymerase chain reaction (RT‐PCR) test to detect SARS‐CoV‐2 variants, followed by whole genome sequencing by Nanopore technology. Samples were retrieved from different sites and stored at −70°C deep freezer, and tests were performed on samples with cycle threshold <30.

**Results:**

Out of 4879 nucleic acid tests, 799 whole genome sequencing had been carried out. Among the stored samples of earlier local transmission, we found the 20B (B.1.1.46) variant predominated in the earlier local transmission period. A slower introduction and circulation of alpha and delta variants were observed compared to global dynamics in 2020 and 2021. Beta or Gamma variants were not detected between November 2020 and September 2021 in Mongolia.

**Conclusions:**

SARS‐CoV‐2 variants of concerns including alpha and delta were delayed in circulation potentially due to public health stringencies in Mongolia. We are sharing our initial experience with whole genome sequencing of SARS‐CoV‐2 from Mongolia, where sequencing data is sparse.

## Graphical abstract

We are sharing our initial experience with whole genome sequencing of severe acute respiratory syndrome coronavirus 2 (SARS‐CoV‐2) from Mongolia, where sequencing data is sparse. We used retrospective analysis of stored biological samples from November 2020 to October 2021 and a variant‐specific real‐time reverse transcription polymerase chain reaction test to detect SARS‐CoV‐2 variants in 4879 samples, followed by whole genome sequencing by Nanopore technology in 799 samples. Among the samples, the 20B (B.1.1.46) variant predominated in the earlier local transmission period. We found slower introduction and circulation of alpha and delta variants in our country compared to global dynamics in 2020 and 2021.

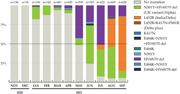

## 1 INTRODUCTION

Coronavirus disease (COVID‐19) has had a profound impact on the world since its emergence in December 2019.^1^, ^2^ The rapid spread of the virus across the globe prompted an urgent need for research into its epidemiology and transmission patterns, particularly for the emergence of severe acute respiratory syndrome coronavirus 2 (SARS‐CoV‐2) variants which had strong impacts on its transmissibility and disease severity.^3^, ^4^ Whole genome sequencing is an essential part of molecular epidemiology and surveillance studies to identify the emerging variants of concern.^5^ However, whole genome sequencing capabilities and global network remain a challenging issue, particularly among developing countries, including Mongolia.^6^ Limited amount of SARS‐CoV‐2 sequencing data were reported to the GISAID (*n* = 781) and Nextstrain (*n* = 17) databases, respectively.^7^, ^8^

Mongolian public health emergency oriented early response measures were one of the excellently orchestrated set of measures among the lower‐ and middle‐income countries (LMICs).^9^ The first confirmed case of COVID‐19 in Mongolia was reported on March 10, 2020, in a French national who had recently traveled to the country.^9^ Since then, the Mongolian government has implemented a range of measures to control the spread of the virus, including strict lockdowns, border closures, and mass testing.^9^, ^10^ These measures allowed to prevent from local transmission until November 2020. A national populational serial serological surveys of SARS‐CoV‐2 confirmed low prevalence setting between late 2020 and early 2021, and rapidly growing rates for seropositivity induced by vaccination and natural infection onwards by mid and late 2021.^10^, ^11^ As of March 17, 2023, Mongolia reported a total of 2136 deaths due COVID‐19 and over 1,007,907 confirmed cases among 3,480,785 population.^12^ Cases continued to decline in 2023, and the country has had relatively lower fatality rates compared to other developing countries.^13^, ^14^ In our study period as of September 30, 2021, Mongolia reported 382,412 confirmed cases and 1241 deaths per 3,409,939 population.^12^ However, due combination of lower confirmed cases of SARS‐CoV‐2 until the second quarter of 2021, and lack of national whole genome sequencing availabilities, systematic knowledge of SARS‐CoV‐2 variants and molecular epidemiology in 2020 and 2021 remained opaque in Mongolia.

Therefore, we conducted extensive SARS‐CoV‐2 molecular epidemiological study in the country. We explored the early molecular epidemiologic patterns of SARS‐CoV‐2 in Mongolia, to gain in depth understanding of SARS‐CoV‐2 transmission in accordance with variants and related factors of epidemic dynamics. Second, we analyzed the agreements between variant determining real‐time reverse transcription polymerase chain reaction (RT‐PCR) and whole genome sequencing methods. We report the molecular patterns of SARS‐CoV‐2 covering the period for early local transmission and national vaccination campaign, and months of initial two waves for SARS‐CoV‐2 in Mongolia, 2020–2021.

## 2 MATERIALS AND METHODS

### 2.1 Study design and timeline

We conducted a nationally representative molecular epidemiologic study of SARS‐CoV‐2 using retrospective analysis of SARS‐CoV‐2 stored biological samples in Mongolia for 2020 and 2021. Our study serves as genomic surveillance study on SARS‐CoV‐2 on variants, since variant testing capacity was introduced later in Mongolia. In the study, samples were retrieved and analyzed between June 2021 and December 2022 for SARS‐CoV‐2 variant analysis and whole genome sequencing. Biological samples collected from November 2020 to October 2021 were included in the study. Initial sampling dates were considered as study timeline.

### 2.2 Study setting

According to the Law on Disaster Protection of Mongolia, clinical and preventive health services for public health emergencies are entirely covered by the government regardless of citizenship and insurance statuses. Study timeline covers free testing strategy period including One‐Door One‐Test mass testing in Ulaanbaatar, and limited availability of real‐time RT‐PCR testing periods coupled with out‐of‐pocket payment testing in February to September, 2021.^15^

During study timelines, nucleic acid amplification testing to detect SARS‐CoV‐2 was used as a means of confirming COVID‐19 throughout the study period, and later supported by rapid antigen test and clinical definition of illness for close contacts of confirmed SARS‐CoV‐2 cases.

Biological samples were consisting from voluntary testing, mass testing, clinical, contact tracing, occupational exposure‐related tests and others. However, we only included real‐time RT‐PCR‐confirmed samples.

### 2.3 Data collection and sites

Earlier the local transmission, the national referral centers’ laboratories were testing all samples from suspected cases, and all SARS‐CoV‐2 positive samples were stored at these sites. RT‐PCR positive, stored samples tested at (i) Clinical Molecular Diagnostic Center, Mongolian National University of Medical Sciences, (ii) National Center for Communicable Diseases, (iii) National Center for Public Health, (iv) National Center for Zoonotic Disease, and (v) Intermed Hospital in Ulaanbaatar city. Rural samples were tested and stored at the above‐mentioned laboratories. Thus we accessed all stored positive samples, where available.

### 2.4 Data sources and variables

We retrieved sociodemographic and residential information from national databases including the Gerege Systems, tandalt.gov.mn, and health.gov.mn databases with proper permission and rights. Data collected from these databases include date of birth, current residence, sex, vaccination dates, vaccine type, sample collection dates for SARS‐CoV‐2 testing date, and others.

### 2.5 Sample collection and storage

Nasopharyngeal swabs in viral transport media and salivary samples were eligible for the analysis. We extracted RNA onsite at sample‐stored laboratories according to StarMag 96 Proprep C protocol. RNA extracts were stored at −70°C deep freezer, the Institute of Biomedical Sciences, Mongolian National University of Medical Sciences for further analysis.

### 2.6 Sample retrieval strategy and required sample size

We retrieved samples from molecular diagnostic center in Mongolia for locally transmitted cases in 2020 and 2021. At first stage of the study, the stored domestically reported samples from nasopharyngeal swabs and saliva from molecular diagnostic laboratories performing real‐time RT‐PCR was performed to detect mutations of SARS‐CoV‐2 spike (S) gene.

We followed the WHO Interim guidance of August 09, 2021 “Guidance for surveillance of SARS‐CoV‐2 Variants”^5^ as guided for sample size calculation. SARS‐CoV‐2 confirmed case numbers were low between November 2020 and February 2021, and therefore monthly confirmed cases were used for reference quantity for variant specific real‐time RT‐PCR. Since locally transmitted cases were growing endemically later, we used the weekly moving average of SARS‐CoV‐2 confirmed cases to calculate the required sample size for SARS‐CoV‐2 variant specific real‐time RT‐PCR testing from March to October, 2021. Details of calculations for sample size are available on Supporting Information S1: Additional file 1. According to the WHO Interim guidance, we used the moving average of weekly confirmed cases in Mongolia. We aimed to analyze recommended sample numbers for weekly variants change of 2.5%–10%, since we did not have systematic information on variants circulated in Mongolia.

### 2.7 Real‐time RT‐PCR protocol

SEEPREP32 (Seegene Inc) was used to extract SARS‐CoV‐2 ribonucleic acid (RNA) from positive samples of confirmed COVID‐19 cases. The StarMag 96 ProPrep C Nucleic Acid Extraction Kit was used in the extraction process. Real‐time RT‐PCR and whole genome sequencing were performed on the extracted RNA samples.

Real‐time RT‐PCR was used to detect S gene mutations including E484K, N501Y, HV69/70 del, L452R, W152C, K417N, K417T, and P681R. The CFX 96 real‐time PCR Detection System (Bio‐Rad, Hercules) was used in conjunction with the Allplex SARS‐CoV‐2 Variants I, II Assay and Novaplex SARS‐CoV‐2 Variants IV Assays.

### 2.8 Whole genome sequencing protocol

In the next stage of the study, a whole genome sequencing analysis of SARS‐CoV‐2 was conducted. The target sample size for sequencing was determined based on the results of the real‐time RT‐PCR for the detection of specific mutations in the S gene and the timeline of infection confirmation. The sequencing sample size target was at least 20% (increased by 10%–20% depending on sample availability) of the sample size for SARS‐CoV‐2 variant‐specific real‐time RT‐PCR tests per month. SARS‐CoV‐2 RNA samples with a cycle threshold (CT) < 30 were selected for whole genome sequencing, with the real‐time RT‐PCR variant proportions maintained at the highest possible level.

The SARS‐CoV‐2 whole genome sequencing analysis was carried out at the Molecular Biology Laboratory, Institute of Biomedical Sciences, Mongolian National University of Medical Sciences, using the MinION Mk1C (Oxford Nanopore Technologies) sequencing device and “PCR tiling of SARS‐CoV‐2 virus‐rapid barcoding and Midnight RT PCR Expansion” protocol. The sequence results were analyzed by EPI2ME Agent, a web‐based software that determined viral genomic variants by Nextstrain clade (Pangolin lineage is additionally provided).

### 2.9 Data analysis and interpretation

Distribution of SARS‐CoV‐2 variants and mutations are presented in percentages (%) of weight in the samples per month or in the total samples. We followed manufacturer's protocol algorithm for assigning SARS‐CoV‐2 variants and mutations per guidance for Allplex SARS‐CoV‐2 Variants I, II Assay, and Novaplex SARS‐CoV‐2 Variants IV Assays (Seegene Inc).

We determined sensitivity, specificity, positive predictive value (PPV), negative predictive values (NPV) rates, and Cohen's *κ* coefficient of Allplex SARS‐CoV‐2 Variants I, II Assay, and Novaplex SARS‐CoV‐2 Variants IV Assays (Seegene Inc) real‐time RT‐PCR kits against the MinION Mk1C (Oxford Nanopore Technologies) whole genome sequencing test as golden standards test, using the IBM SPSS Statistics 26.0.

## 3 RESULTS

### 3.1 Demography and sample characteristics

Table 1 demonstrates the demographic and immunization statuses of participants and virological characteristics according to real‐time RT‐PCR testing. Adults aged 30–39 had the highest representation, while seniors had lowest among samples. More females were included in the analysis (Table 1). Across the sample collection timelines, majority (80%) of the individuals were vaccinated by at least one dose of SARS‐CoV‐2 vaccines, while 57% had statuses of vaccinated before SARS‐CoV‐2 infection in Mongolia (Table 1).

**Table 1:** Study sample characteristics and confirmed cases in Mongolia.

		Study samples		Confirmed cases of the population^a^	
		*n*	%	*n*	%
Age group	0–9	450	9.2	99,030	25.9
	10–19	453	9.3		
	20–29	944	19.3	64,637	16.9
	30–39	1175	24.1	86,420	22.6
	40–49	764	15.7	58,141	15.2
	50–59	566	11.6	40,562	10.6
	60–69	236	4.8	21,454	5.6
	70–79	87	1.8	11,901	3.1
	≤80	54	1.1		
	Unknown	150	3.1		
Sex	Male	2133	43.7	170,819	44.7
	Female	2596	53.2	211,326	55.3
	Unknown	150	3.1		
Vaccination before severe acute respiratory syndrome coronavirus 2 infection	Vaccinated	2756	56.5		
	Refused	1090	22.3		
	Unvaccinated	992	20.3		
	Unknown	41	0.8		
Region of residence	Ulaanbaatar	3114	63.8	209,183	54.7
	Khangai region	1038	21.3	54,584	14.3
	Central region	341	7.0	43,765	14.1
	Eastern region	70	1.4	25,231	6.6
	Western region	50	1.0	49,382	12.9
	Unknown	266	5.5		
Sample type	Nasopharyngeal swab	4238	86.9		
	Saliva	641	13.1		

^a^ The Ministry of Health of Mongolia, coronavirus disease 2019 (COVID‐19) daily report for September 30, 2021

**Figure 1:**
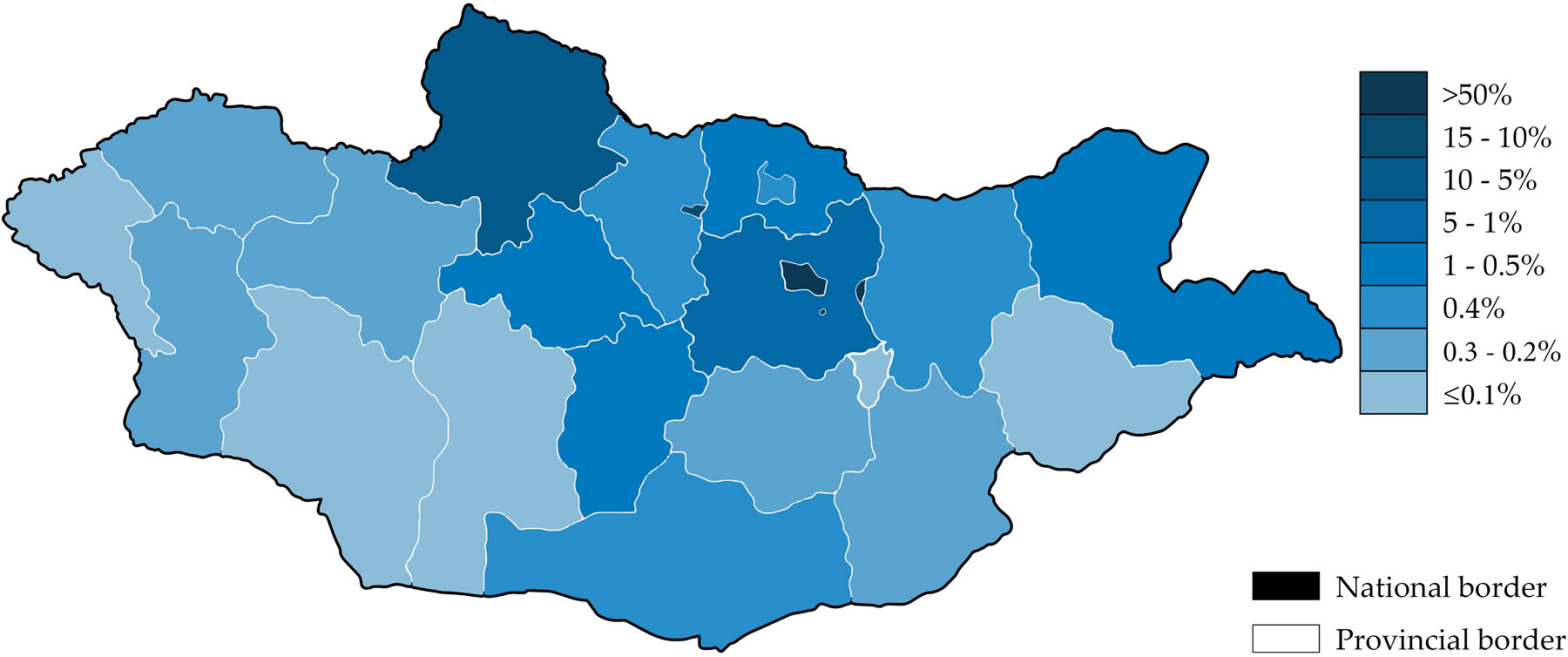
Geolocational information of participants' current residency in Mongolia, 2020–2021.

Predominantly, the nasopharyngeal swab samples were analyzed, whilst only 13% were salivary samples. Majority of the samples collected were from Ulaanbaatar city residents, followed by Khangai and Central region residents of Mongolia (Table 1). The distribution of current residential location of hosts are illustrated in Figure 1, by provincial levels of Mongolia. A total of 4879 samples were included in the analysis for SARS‐CoV‐2 variant specific real‐time RT‐PCR and more.

### 3.2 Real‐time RT‐PCR findings and timeline of SARS‐CoV‐2 variants in circulation

During study timeline, ancestral SARS‐CoV‐2 strain without S gene mutation represented the highest proportions (43%), followed by alpha variant (21%), HV69/70 del (20%), and delta variants (15%) of SARS‐CoV‐2. Combinations of S gene mutations and others represented remaining smaller proportions (Table 2).

**Table 2:** Summary of real‐time reverse transcription polymerase chain reaction (RT‐PCR) detected severe acute respiratory syndrome coronavirus 2 mutation findings in Mongolia, 2020–2021.

		*n*	%
Interpretation of variant specific real‐time RT‐PCR	No mutation	2107	43.2
	Alpha	1040	21.3
	HV69/70 del	952	19.5
	Delta	706	14.5
	N501Y	34	0.7
	Delta Plus	5	0.1
	Beta or Gamma	4	0.1
	E484K + HV69/70 del	2	0.0
	E484K	1	0.0

**Figure 2:**
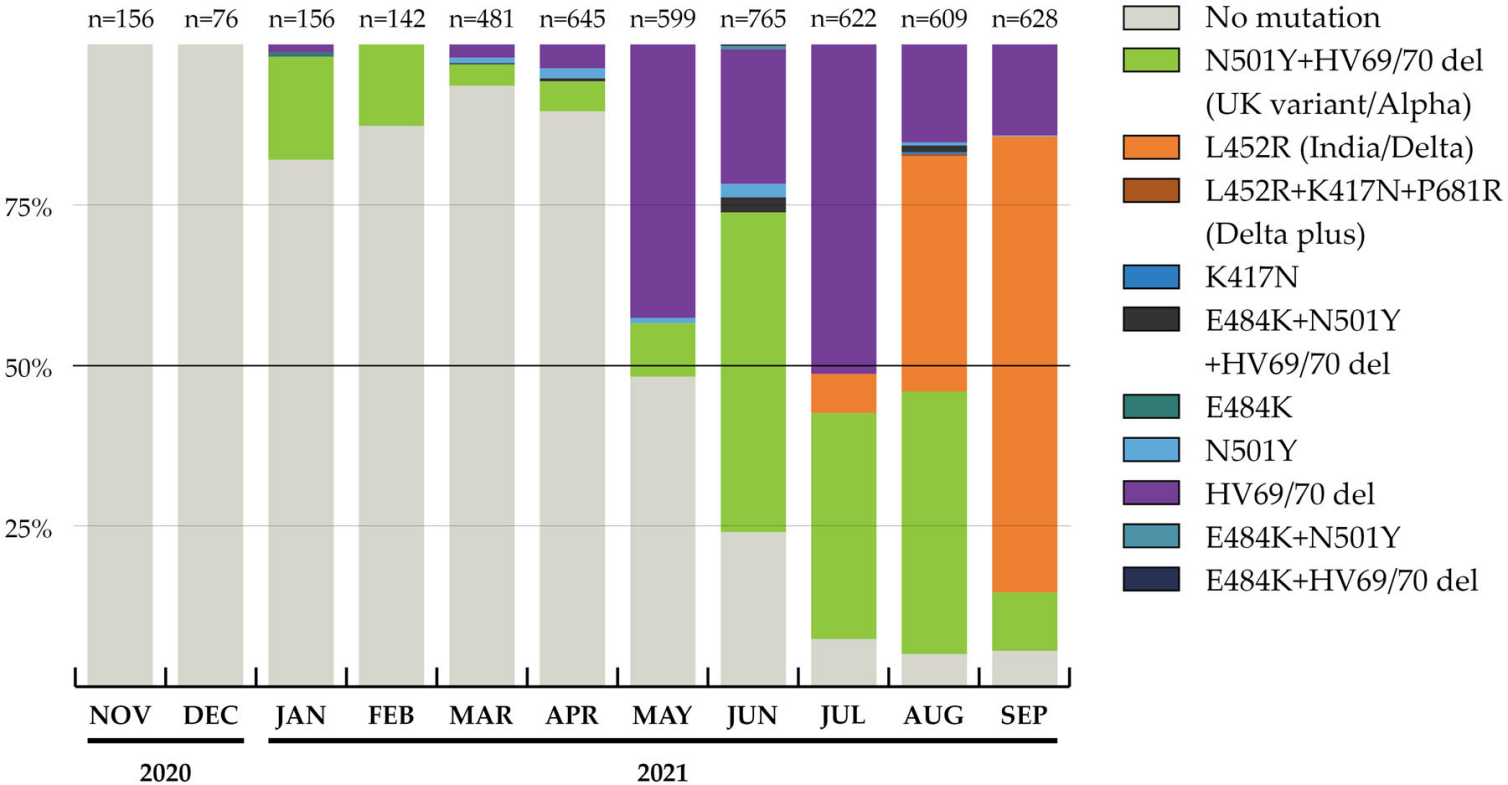
Monthly distribution of severe acute respiratory syndrome coronavirus 2 variants and mutations in Mongolia, 2020–2021, by real‐time reverse transcription polymerase chain reaction detection.

Variant detection results are presented monthly in Figure 2, according to real‐time RT‐PCR Seegene Inc) Allplex SARS‐CoV‐2 Variants I, II Assay, and Novaplex SARS‐CoV‐2 Variants IV Assays protocols. Among analyzed samples, December 2020 had the lowest sample size while June 2021 had the highest sample representation. Across the samples, the ancestral original variant of SARS‐CoV‐2 predominated until May 2021 in Mongolia by real‐time RT‐PCR testing. The alpha variant of SARS‐CoV‐2 started circulating in January 2021 and peaked in June 2021. The HV69/70 deletion had a similar pattern of distribution as the alpha variant, with a peak in July 2021. Meanwhile, the delta variant of SARS‐CoV‐2 emerged from July 2021 and peaked in September 2021, representing over two‐thirds of tested samples. In addition, Figure 2 shows monthly confirmed cases of SARS‐CoV‐2 in Mongolia and coverage rate in our study.

### 3.3 SARS‐CoV‐2 whole genome sequence results

**Figure 3:**
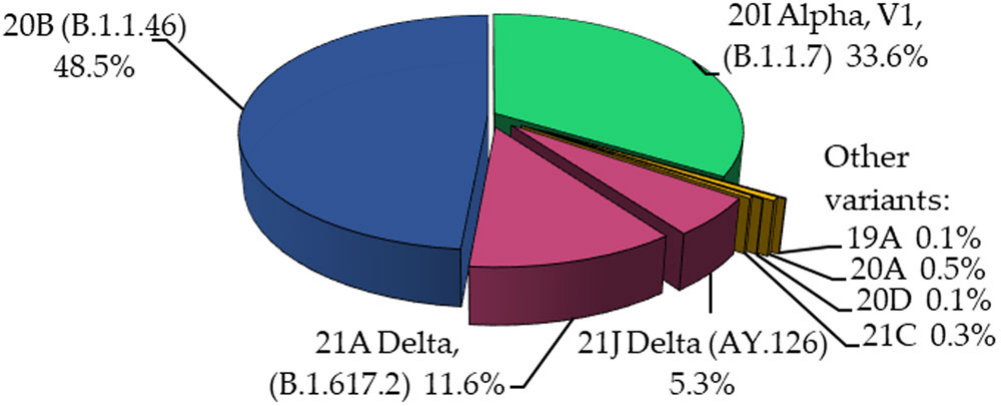
Proportions of severe acute respiratory syndrome coronavirus 2 variants by Nextclades (Pangolin lineage) using whole genome sequencing in Mongolia, 2020–2021.

The whole genome sequencing results are summarized in Figure 3, (*n* = 799). Among total sequenced samples, SARS‐CoV‐2 ancestral variant 20B (B.1.1.46) were highest (49%) proportions. Secondly, SARS‐CoV‐2 20I Alpha, V1, (B.1.1.7) were at 34%. 21A (B.1.617.2) and 21J (AY.126) Delta variants were confirmed at 12% and 5%, respectively in Mongolia, by whole genome sequencing. Remaining proportions were variety of SARS‐CoV‐2 nextclades (Figure 3).

### 3.4 Validity of SARS‐CoV‐2 variant specific real‐time RT‐PCR tests

SARS‐CoV‐2 test agreements of variant specific real‐time RT‐PCR testing compared against the whole genome sequencing are summarized in Table 3, according to test kit designs of Variant 1 and Variant 2 for emerging SARS‐CoV‐2 variants. The agreements of real‐time RT‐PCR testing differed between variants, respectively.

**Table 3:** Agreements between severe acute respiratory syndrome coronavirus 2 (SARS‐CoV‐2) variant determining real‐time reverse transcription polymerase chain reaction and whole genome sequencing, Mongolia 2020–2021.

	*N*	Kappa	St. error	*p*‐Value
Alpha	799	0.490	0.033	<.001
Delta	799	0.973	0.011	<.001
Others and no mutation^a^	799	0.591	0.027	<.001

^a^ Combination of 20B (B.1.1.46) and variety of SARS‐CoV‐2 variants other than alpha and delta.

Table 4 presents validity of variant specific real‐time RT‐PCR testing. Alpha variant of SARS‐CoV‐2 had the lowest sensitivity at 50%, while the test had very high specificity (95%). Delta variant had both very high rates of sensitivity and specificity. In overall the real‐time RT‐PCR testing had 99% sensitivity and 99% specificity for detecting the delta variant. Meanwhile, the sensitivity for ancestral SARS‐CoV‐2 and other mutation was at 95% and 82% for Variant 1 and Variant 2 assays, respectively.

**Table 4:** Variant specific real‐time reverse transcription polymerase chain reaction (RT‐PCR) testing accuracy against whole genome severe acute respiratory syndrome coronavirus 2 (SARS‐CoV‐2) sequencing in Mongolia, 2020–2021.

	Total
*Alpha*	
Sensitivity	49.4% (43.5–55.4)
Specificity	94.7% (92.6–96.4)
Positive predictive value (PPV)	82.6% (76.2–87.9)
Negative predictive value (NPV)	78.7% (75.4–81.8)
*Delta*	
Sensitivity	98.5% (95.3–99.7)
Specificity	99.4% (98.6–99.8)
PPV	97.1% (93.2–99.0)
NPV	99.7% (99.0–99.9)
*Others and no mutation* a	
Sensitivity	92.7% (89.8–94.9)
Specificity	66.6% (61.9–71.1)
PPV	73.1% (69.1–76.8)
NPV	90.3% (86.5–93.2)

^a^ Combination of 20B (B.1.1.46) and variety of SARS‐CoV‐2 variants other than alpha and delta.

## 4 DISCUSSION

Our findings were from one of the first systematically recruited, retrospectively designed, and nationally representative genomic surveillance studies for SARS‐CoV‐2 variants in Mongolia, although we analyzed only 1.3% of positive samples. The study covered a period of 11 months in 2020 and 2021, starting from the notification of domestic transmission in November 2020. We covered the most of testing strategy period whereas the real‐time RT‐PCR tests were available for testing SARS‐CoV‐2 for free of charge in Mongolia.^16^, ^17^ Later in 2021 with the rapid increase in the number of cases, clinical symptoms and contact history, and rapid antigen test were recommended for SARS‐CoV‐2 diagnosis.^18^

We found that the SARS‐CoV‐2 20B variant, as determined by Nextstrain, was the most dominant followed by the Alpha and Delta variants of SARS‐CoV‐2. The distribution and evolution patterns of SARS‐CoV‐2 variants were similar to the global trends.^19^

During the first few months of local transmission of SARS‐CoV‐2 in Mongolia, the country had implemented stringent containment policy measures, including lockdowns, quarantine of incoming travelers, mandatory mask‐wearing, and the cancellation of commercial flights. The country gradually reduced these measures following the introduction of the SARS‐CoV‐2 vaccination campaign in February 2021.^9^, ^10^ The predominance of the SARS‐CoV‐2 20B variant, as determined by Nextstrain, is supported by the scarcity of S gene mutations on real‐time RT‐PCR tests until May 2021.

The proportions of the Alpha variant among SARS‐CoV‐2 variants in Mongolia were lower compared to neighboring Russian Federation and other countries that practiced less stringent policy measures, including the Philippines, United States, United Kingdom, and Japan. As of May 2021, the percentage of Alpha variant among sequenced samples in these countries ranged from 27% to 98%, while in Mongolia, fewer than 10% of real‐time RT‐PCR tests were assigned as Alpha variant, followed by a five‐fold increase in June 2021.^12^

Lower alpha variant distribution until June 2021 may be due to several reasons and associated factors. Until the introduction of SARS‐CoV‐2 vaccines, incoming travelers were quarantined and restrictions to public gathering continued. This prolonged isolation period could have led to slower spread of alpha variant until early 2021. Another potential factor of low representation could be due relatively low sensitivity rate of real‐time RT‐PCR testing in our study, for alpha variant. Sequencing was recommended by the European Centre for Disease Prevention and Control for monitoring and molecular characterizing of the Alpha variant, due multiple random mutations in S gene in the United Kingdom.^20^ Distribution of SARS‐CoV‐2 variants in Mongolia were similar to the patterns observed in the Russian Federation, potentially due to first introduction from northern border with Russian Federation and earlier re‐opening of cross border travels.

During the study period, we did not detect any Gamma or Beta variants of SARS‐CoV‐2 among all the samples we tested. These variants were predominantly reported in regions and countries such as Brazil and South Africa, respectively.^12^ Given the lower travel history to these countries and the vast geographical distance, Mongolia may not have experienced an outbreak of Beta or Gamma variants, at least during the study period.

In July 2021, first cases of the delta variant were detected in our study. Meanwhile across the globe, the proportion of delta variant had been dominated in neighboring and regional countries^12^ including the Russian Federation,^21^ South Korea,^22^ and Vietnam.^23^ In Mongolia, the Delta variant had apparently become dominant by September 2021, following the loosening of restrictive measures, including the resumption of commercial flights and presidential election campaigns. These patterns suggest a relatively slower introduction of globally circulating variants.^12^

Nanopore sequencing is being used worldwide to quickly sequence and analyze SARS‐CoV‐2 viral genomes.^24^ Rapid data sharing enables genomic epidemiological analysis, a key part of the global public health response to the COVID‐19 pandemic.^25^ Nanopore technology has been used to rapidly sequence the emerging and reemerging pathogens including Zika, Ebola, and other pathogens in multiple outbreaks, where surveillance and genomic investigation capacities are limited.^26^, ^27^, ^28^, ^29^, ^30^ Oxford Nanopore has developed workflows for rapid preparation and sequencing of SARS‐CoV‐2 whole genomes, with starter packs that are affordable for LMICs. The ARTIC bioinformatics pipeline is implemented in EPI2ME Labs Workflow, providing an easy‐to‐follow tutorial for less experienced scientists and healthcare professionals. The cloud‐based EPI2ME Workflow enables real‐time analysis without prior bioinformatics experience.^24^ Mongolia had limited capacity and experiences of next‐generation sequencing in outbreak biomonitoring and genomic bioinformatic analytics. In developing countries setting, conventional next‐generation sequences are commonly challenging and requires extensive bioinformatics expertise. Moreover, the Oxford MinION, a third‐generation nanopore‐based platform has been used in several LMICs including India, Bangladesh, Indonesia, and others for SARS‐CoV‐2 nationwide surveillance studies.^31^, ^32^, ^33^, ^34^, ^35^, ^36^, ^37^, ^38^ This study represents a pioneering genomic surveillance effort, involving systematic sampling in Mongolia for respiratory viral pathogens. In Armenia, a molecular surveillance study of SARS‐CoV‐2 was conducted using nanopore sequencing, in addition to the Illumina sequencing platform.^39^ This study demonstrates the feasibility of using Nanopore next‐generation sequencing in LMICs, and promotes its adoption in these settings.

This study had several significant scientific and global public health implications. First, we were able to timely notify policymakers about the local transmission of the delta variant and provide key evidence on its emergence in Mongolia.^40^ Second, our two‐step approach using variant‐specific real‐time PCR‐based molecular detection and whole genome sequencing proved to be an efficient method in a resource‐limited setting.^41^ Last, our study provides nationally representative findings from a previously less explored geographic location in the world for SARS‐CoV‐2 molecular epidemiology.

Mongolia has rich wildlife and human interactions in rural parts of the country. In combination with the traditional animal husbandry rooted nomadic lifestyle, the country experiences relatively common zoonotic diseases and outbreaks including bubonic plague, tickborne diseases, and anthrax.^42^ In future outbreaks and wildlife surveillance studies, Nanopore technologies can be utilized for whole genome sequencing and early detection of potential pandemics in field epidemiology works. Previously, Middle East respiratory syndrome coronavirus surveillance was conducted among Mongolian camels related to wildlife surveillance, however resulted in null findings with real‐time RT‐PCR.^43^ Field epidemiological studies in wildlife could utilize similar approaches for SARS‐CoV‐2 and other pathogens for wild animals and livestock samples in Mongolia rural areas upon emergence.

Our study had several limitations. The Mongolian national laboratory network did not have a strong SARS‐CoV‐2 variant surveillance system for detection at a nationwide level, which may have resulted in potential selection biases, particularly in the earlier months of SARS‐CoV‐2 local transmission. Additionally, due to the exponential growth of local transmission in 2021 across the country, our samples may not represent the geographical distribution of local cases in Mongolia. Finally, our study may contain recall bias as the laboratory information system (LIS) is not established nationally and currently does not have interoperability functions at several national referral hospitals and centers. Due to the absence of LIS in Mongolia, we were not able to retrieve host demography and sample storage archives for all positive samples.

We are sharing our first experiences of next‐generation sequencing in Mongolia, where we have sequenced nearly 800 whole genomes of SARS‐CoV‐2. In the future, whole genome sequencing, particularly using next‐generation sequencing platforms, should be utilized for identifying emerging and re‐emerging pathogens. Our study represents the first large‐scale next‐generation sequencing experience in Mongolia, and the lessons learned from this study should be noted for the next pandemic emergence or the variants of concern for the ongoing COVID‐19 pandemic. We should also contemplate the ease and operability of Nanopore sequencing and other next‐generation sequencing methods to be utilized in field epidemiology. The global‐south nations have similar rich wildlife exposure and rural settings, as described in our study.^44^

## 5 CONCLUSIONS

In this study, we observed several unique characteristics of SARS‐CoV‐2 variants dynamics, including the slower introduction of the alpha variant and the rapid expansion of the delta variant in the second half of 2021 in Mongolia. With the emergence of SARS‐CoV‐2, Mongolia had carried out a large‐scale next‐generation sequencing tests for the first time given to the limited resources and capacity. Furthermore, capacities need to be expanded to institutionalize molecular surveillance in the country. The SARS‐CoV‐2 variant‐specific real‐time RT‐PCR test demonstrated very high sensitivity and specificity rates at least for the delta variant in our experience.

## AUTHOR CONTRIBUTIONS

**Munkhtuya Erendereg**: Conceptualization; data curation; formal analysis; investigation; methodology; software; writing—original draft. **Suvd Tumurbaatar**: Formal analysis; funding acquisition; investigation; methodology; software; validation. **Otgonjargal Byambaa**: Formal analysis; software. **Gerelmaa Enebish**: Formal analysis. **Natsagdorj Burged**: Formal analysis. **Tungalag Khurelsukh**: Formal analysis. **Nomin‐Erdene Baatar**: Formal analysis. **Badmaarag Munkhjin**: Data curation; software. **Jargaltulga Ulziijargal**: Data curation; writing—original draft. **Anuujin Gantumur**: Formal analysis. **Oyunbaatar Altanbayar**: Formal analysis. **Ochbadrakh Batjargal**: Software. **Delgermurun Altangerel**: Validation. **Khosbayar Tulgaa**: Validation. **Sarangua Ganbold**: Data curation. **Odgerel Tundev**: Validation. **Sarantsetseg Jigjidsuren**: Data curation. **Tsogbadrakh Nyamdorj**: Project administration. **Naranzul Tsedenbal**: Project administration. **Bumdelger Batmunkh**: Formal analysis; project administration. **Baigalmaa Jantsansengee**: Project administration. **Battur Lkhagvaa**: Project administration. **Bilegtsaikhan Tsolmon**: Project administration. **Oyunsuren Enebish**: Supervision. **Erdembileg Tsevegmid**: Supervision. **Enkhbold Sereejav**: Supervision. **Khurelbaatar Nyamdavaa**: Conceptualization; project administration; Supervision; writing—review and editing. **Ryenchindorj Erkhembayar**: Data curation; visualization; writing—original draft; writing—review and editing. **Battogtokh Chimeddorj**: Conceptualization; funding acquisition; methodology; project administration; resources; supervision; writing—original draft; writing—review and editing.

## CONFLICT OF INTEREST STATEMENT

The authors declare no conflict of interest.

## ETHICS STATEMENT

The study was conducted in accordance with the Declaration of Helsinki, and approved by the and Ethics Committee of Ministry of Health, Mongolia (No 173, date of approval: July 8, 2020) and Ethics Committee of Mongolian National University of Medical Sciences (No 2021/3‐07, date of approval: June 4, 2021). Both committees approved informed consent waiver for stored biological sample analysis. Official approval and permission to use specimens were obtained from each organization.

## Supporting information

Supporting information.

## Data Availability

The data presented in this study are available on request from the corresponding author. The most amount of the sequencing result is shared publicly at https://gisaid.org/hcov19-variants/.
