# Supplementary material for: Molecular epidemiology of SARS‐CoV‐2 in Mongolia, first experience with nanopore sequencing in lower‐ and middle‐income countries setting
Source: Immun Inflamm Dis. 2023 Dec 13;11(12):e1095. doi: 10.1002/iid3.1095 (PMC10716720; doi:10.1002/iid3.1095)
Supplement: Supplementary file 1 — Supporting information. [file IID3-11-e1095-s001.docx]

***Appendices***

Sample size calculation for real-time RT-PCR and sequencing

| Month/week | 7- day moving average | New cases | Sample size | |
| --- | --- | --- | --- | --- |
|  |  |  | **real-time RT-PCR** | **Sequencing** |
| Nov, 2020 |  |  |  |  |
| 11/7/2028 | 2 | 14 |  |  |
| 11/14/2020 | 8 | 56 |  |  |
| 11/21/2020 | 21 | 147 |  |  |
| 11/28/2020 | 26 | 182 |  |  |
| total |  | **399** | **103** | **21** |
| Dec, 2020 |  |  |  |  |
| 12/5/2020 | 14 | 98 |  |  |
| 12/12/2020 | 10 | 70 |  |  |
| 12/19/2020 | 5 | 35 |  |  |
| 12/26/2020 | 19 | 133 |  |  |
| 1/2/2021 | 23 | 161 |  |  |
| total |  | **497** | **103** | **21** |
| Jan, 2021 |  |  |  |  |
| 1/9/2021 | 24 | 168 |  |  |
| 1/16/2021 | 15 | 105 |  |  |
| 1/23/2021 | 14 | 98 |  |  |
| 1/30/2021 | 19 | 133 |  |  |
| total |  | **504** | **115** | **23** |
| Feb, 2021 |  |  |  |  |
| 2/6/2021 | 35 | 245 |  |  |
| 2/13/2021 | 43 | 301 |  |  |
| 2/20/2021 | 42 | 294 |  |  |
| 2/27/2021 | 40 | 280 |  |  |
| total |  | **1120** | **123** | **25** |
| Mar, 2021 |  |  |  |  |
| 3/6/2021 | 42 | 294 | 103 |  |
| 3/13/2021 | 96 | 672 | 103 |  |
| 3/20/2021 | 139 | 973 | 103 |  |
| 3/27/2021 | 269 | 1883 | 123 |  |
| total |  | **3822** | **432** | **86** |
| Apr, 2021 |  |  |  |  |
| 4/3/2021 | 415 | 2905 | 126 |  |
| 4/10/2021 | 655 | 4585 | 126 |  |
| 4/17/2021 | 924 | 6468 | 128 |  |
| 4/24/2021 | 1223 | 8561 | 128 |  |
| 5/1/2021 | 1152 | 8064 | 128 |  |
| total |  | **30583** | **636** | **127** |
| May, 2021 |  |  |  |  |
| 5/8/2021 | 961 | 6727 | 128 |  |
| 5/15/2021 | 584 | 4088 | 126 |  |
| 5/22/2021 | 548 | 3836 | 126 |  |
| 5/29/2021 | 670 | 4690 | 126 |  |
| total |  | **19341** | **506** | **101** |
| Jun, 2021 |  |  |  |  |
| 6/5/2021 | 1051 | 7357 | 128 |  |
| 6/12/2021 | 1417 | 9919 | 128 |  |
| 6/19/2021 | 2465 | 17255 | 129 |  |
| 6/26/2021 | 2301 | 16107 | 129 |  |
| total |  | **50638** | **514** | **103** |
| Jul, 2021 |  |  |  |  |
| 7/3/2021 | 2211 | 15477 | 129 |  |
| 7/10/2021 | 1902 | 13314 | 129 |  |
| 7/17/2021 | 1391 | 9737 | 128 |  |
| 7/24/2021 | 1348 | 9436 | 128 |  |
| 7/31/2021 | 1417 | 9919 | 128 |  |
| total |  | **57883** | **642** | **128** |
| Aug, 2021 |  |  |  |  |
| 8/7/2021 | 1198 | 8386 | 128 |  |
| 8/14/2021 | 1109 | 7763 | 128 |  |
| 8/21/2021 | 1515 | 10605 | 129 |  |
| 8/28/2021 | 2108 | 14756 | 129 |  |
| total |  | **41510** | **514** | **103** |
| Sep, 2021 |  |  |  |  |
| 9/4/2021 | 3176 | 22232 | 129 |  |
| 9/11/2021 | 4619 | 32333 | 129 |  |
| 9/18/2021 | 5092 | 35644 | 129 |  |
| 9/25/2021 | 7491 | 52437 | 129 |  |
| 10/2/2021 | 6845 | 33201§ | 129 |  |
| total |  | **175847** | **645** | **129** |
| Total |  | **382145** | **4333** | **867** |
| Sampling error 10%-20% | |  | **433-867** | **87-173** |
| TOTAL SAMPLE SIZE | |  | **4766-5200** | **954-1040** |

§ New cases of 01-02, October were excluded from total week cases due calendrical month shifts
